# Effect of Implant Macro-Design and Magnetodynamic Surgical Preparation on Primary Implant Stability: An In Vitro Investigation

**DOI:** 10.3390/dj11100227

**Published:** 2023-09-24

**Authors:** Alessandro Antonelli, Selene Barone, Ferdinando Attanasio, Marianna Salviati, Maria Giulia Cerra, Elena Calabria, Francesco Bennardo, Amerigo Giudice

**Affiliations:** School of Dentistry, Department of Health Sciences, Magna Graecia University of Catanzaro, 88100 Catanzaro, Italy; alessandro.antonelli@unicz.it (A.A.); selene.barone@unicz.it (S.B.); ferdinando.attanasio@gmail.com (F.A.); marianna.salviati@studenti.unicz.it (M.S.); mariagiulia.cerra@studenti.unicz.it (M.G.C.); calabriaelena92@gmail.com (E.C.); a.giudice@unicz.it (A.G.)

**Keywords:** implant macro-design, magnetodynamic osteotomy, primary implant stability, insertion torque, resonance frequency analysis, magnetic mallet, implant site preparation

## Abstract

Background: Macro-geometry and surgical implant site preparation are two of the main factors influencing implant stability and potentially determining loading protocol. The purpose of this study was to assess the initial stability of various implant macro-designs using both magnetodynamic and traditional osteotomy techniques in low-density bone. The parameters examined included peak insertion torque (PIT), implant stability quotient (ISQ), and peak removal torque (PRT). Methods: Four groups of 34 implants each were identified in accordance with the surgery and implant shape: T5 group (*Five* implant and osteotomy using drills); M5 group (*Five* implant and magnetodynamic osteotomy using Magnetic Mallet); TT group (*TiSmart* implant and osteotomy with drills); and MT group (*TiSmart* implant and magnetodynamic osteotomy). Every implant was placed into a low-density bone animal model and scanned using CBCT. The PIT and PRT were digitally measured in Newton-centimeters (Ncm) using a torque gauge device. The ISQ was analyzed by conducting resonance frequency analysis. Results: The PIT values were 25.04 ± 4.4 Ncm for T5, 30.62 ± 3.81 Ncm for M5, 30 ± 3.74 Ncm for TT, and 32.05 ± 3.55 Ncm for MT. The average ISQ values were 68.11 ± 3.86 for T5, 71.41 ± 3.69 for M5, 70.88 ± 3.08 for TT, and 73 ± 3.5 for MT. The PRT values were 16.47 ± 4.56 Ncm for T5, 26.02 ± 4.03 Ncm for M5, 23.91 ± 3.28 Ncm for TT, and 26.93 ± 3.96 Ncm for MT. Based on our data analysis using a t-test with α = 0.05, significant differences in PIT were observed between TT and T5 (*p* < 0.0001), M5 and T5 (*p* < 0.0001), and MT and TT (*p* = 0.02). Significant differences in the ISQ were found between TT and T5 (*p* = 0.001), M5 and T5 (*p* < 0.001), and MT and TT (*p* = 0.01). The PRT also exhibited significant differences between TT and T5, M5 and T5, and MT and TT (*p* < 0.0001). Conclusion: Our data showed favorable primary implant stability (PS) values for both implant macro-geometries. Furthermore, the magnetodynamic preparation technique appears to be more effective in achieving higher PS values in low-density bone.

## 1. Introduction

Oral implantology is a predictable and foreseeable approach for restoring edentulous spaces [1]. The success of implant rehabilitation is based on the ability to achieve the biological phenomenon of osseointegration of the fixtures [2]. The key to obtaining this process is ensuring sufficient stability of the implant, which is crucial in preventing harmful small movements that may result in the formation of fibrous tissue around the implant during the healing process, ultimately leading to implant failure [3]. For this reason, good primary stability (PS) immediately after implant placement is a prerequisite to avoid micromovements during the healing and osseointegration phases of the fixtures [4]. Multiple factors, including the macro-design of the implant, the surgical technique employed, and the quantity and quality of the bone, have an impact on the initial stability of the implant [5,6]. Specifically, when planning for implant-supported prosthetic rehabilitation, the selection of the implant design and the surgery for creating the osteotomy must be tailored to the individual clinical situation and the condition of the surrounding bone. Several clinicians have examined the macro-geometry of implants in order to achieve optimal outcomes in terms of primary implant stability (PS) [7]. In particular, many researchers have shown how the design and properties of the implant threads have a significant impact on the primary implant stability, especially in cases of low-density bone [8,9,10]. In addition to the implant’s shape, the research demonstrated that the type of osteotomy procedure can contribute positively to the modification of the bone surrounding the implant, influencing PS [11]. Due to the frequent lack of PS in the posterior areas of the jaws, many surgical protocols have been developed to enhance the stability of implants [12]. While bone drilling is the main surgical procedure for implant site preparation, it does not consistently achieve adequate PS [13]. This protocol is particularly effective in soft bone where the implant bed is intentionally under-prepared, resulting in improved PS [14]. Alternatively, other surgical techniques have been suggested to enhance the density of the bone surrounding the implant, such as manual bone compaction, osseodensification using burs, or utilizing piezoelectric preparation [15]. Recently, several authors have explored the use of magnetodynamic technology in implant rehabilitation. Exploiting the electromagnetic effect, this technology applies controlled forces to the surgical site within a short moment of impact. These procedures are considered safe for both patients and surgeons due to the precise control and consistency of the applied pressures [16]. The Magnetic Mallet (MM) device employs magnetodynamic technology and consists of a handpiece that is connected to a central management system. The handpiece generates a shockwave at its tip with varying intensities, delivering forces through precise timing (80 μs impact) in accordance with different surgical techniques [16]. This device can utilize various attachments, such as periotomes, osteotomes, and ridge expanders, for specific applications.

This in vitro study aimed to evaluate and compare the primary stability of two different implant macro-designs by evaluating traditional and magnetodynamic surgical approaches in low-density bone. The null hypothesis was no different between the two implant designs in terms of peak insertion torque (PIT), implant stability quotient (ISQ), and peak removal torque (PRT).

## 2. Materials and Methods

Ethical approval was not necessary for the current study, as it involved in vitro experimentation on bone ribs, and no harm or euthanasia of animals was involved throughout the study.

### 2.1. Sample Selection

An experimental ex vivo study model was designed using unfrozen pig ribs selected by CBCT analysis following the inclusion criteria: bone density < 750 Hounsfield units (HU) [17]. This parameter was selected because this part was characterized by a low proportion of bony cortical and in order to simulate a low-density bone area. For each individual rib, implants were placed following the order of the groups considered in this study. All groups analyzed in the study were tested on each individual rib.

### 2.2. Implant Geometry Description

**Figure 1:**
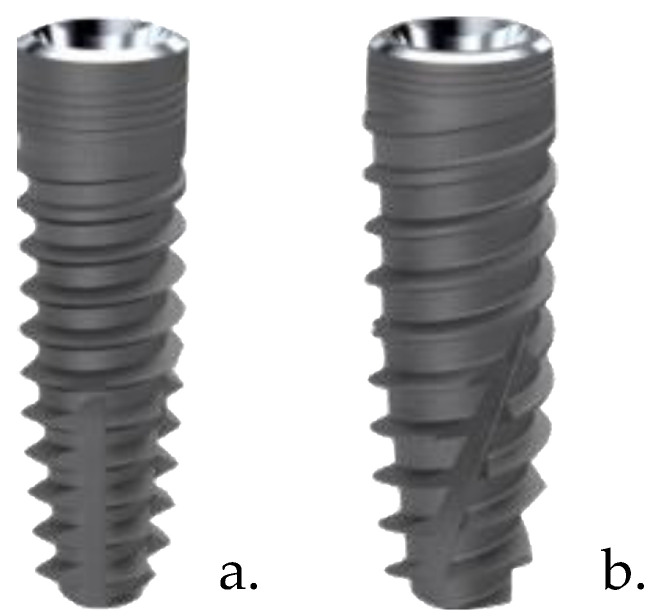
‘Five’ (**a**) and ‘TiSmart’ (**b**) (Leader Medica, Padua) implant macro-design.

Two different implant designs were tested (Figure 1): ‘Five’ Ø4.1 × 10 mm and ‘TiSmart’ Ø4.0 × 10 mm (Leader Medica SRL, Padua, Italy).

- ‘Five’ implants are made of grade 4 titanium (99% pure) manufactured entirely by an industrial process known as aluminum-free processing, which ensures the absence, even of trace amounts, of aluminum on the entire surface of the implant. The implant body is cylindrical in the coronal part, conical in the central and apical part; the implant design is characterized by variable geometry coils (0.4 mm in the coronal part and 0.5 mm in the apical part) with a constant pitch of 1.35 mm and an angle of 60° between the coils. The apical incisions make the implant self-tapping, and microthreading in the coronal part is intended to reduce stress on the cortical portion of the bone.
- ‘TiSmart’ implants are made of grade 4 titanium (99% pure) manufactured entirely by an industrial process known as aluminum-free processing. The implant body is characterized by a coronal part with an interior 6-degree angle, whereas the central part and apex are truncated cone-shaped; threads have a variable geometry, deeper in the “cutting” apex profile and “flat” in the coronal area (0.5 mm in the apical part with a constant pitch of 1.35 mm). The implant’s shape is characterized by three apical incisions, making the fixture “self-tapping” with a rounded apex. The micro threading in the coronal part has the role of minimizing the stress on the cortical bone.

Both implants feature an acid-etched surface treatment without any sandblasting procedure. This treatment is capable of achieving a surface roughness Ra = 1.3 μm.

### 2.3. Experimental Osteotomy Procedures

Two different implant site preparation procedures were performed: a traditional surgical technique with drills and a magnetodynamic osteotomy (Magnetic Mallet^®^-Osseotouch, Gallarate, Italy). According to surgical osteotomy and implant shape, four groups of 34 implants each were identified: T5 group (traditional technique and ‘Five’ implant), M5 group (magnetodynamic technique and ‘Five’ implant), TT group (traditional technique and ‘TiSmart’ implant), and MT (magnetodynamic technique and ‘TiSmart’ implant).

**Figure 2:**
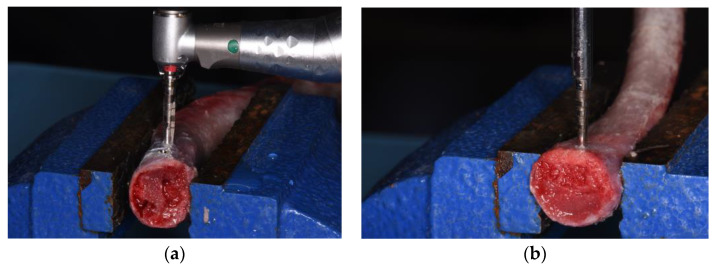
Traditional implant site preparation (**a**). Magnetodynamic implant site preparation (**b**).

The traditional implant site preparation was performed with drills of increasing diameter following the manufacturer’s protocols for both implant geometries (Figure 2a).

The magnetodynamic osteotomy was performed using the MM handpiece with dedicated tips (Osseotouch, Gallarate, VA, Italy); surgical procedures started using a pilot drill and the sequence of tips of increasing diameter for both implant geometries (100-P–160–200–230) (Figure 2b). This surgical approach makes use of electromagnetic impact, which allows for a high intensity and very short duration of impact force to achieve plastic deformation of the bone. The progressive advancement of the tips makes it possible to perform the osteotomy for the implant site preparation.

A single experienced clinician performed all surgical procedures of implant site preparation and fixture placement.

### 2.4. Study Variables

The primary predictor variable in this study was the implant macro-design (‘Five’ and ‘TiSmart’). Subsequently, the influence of the two different surgical techniques (traditional and magnetodynamic osteotomy) was analyzed on each implant design.

Primary stability was assessed by means of the following outcome variables:

- Peak insertion torque (PIT/Ncm);
- Implant stability quotient (ISQ);
- Peak removal torque (PRT)/Ncm.

### 2.5. Outcomes Recording

- Peak insertion torque (PIT)/Ncm

**Figure 3:**
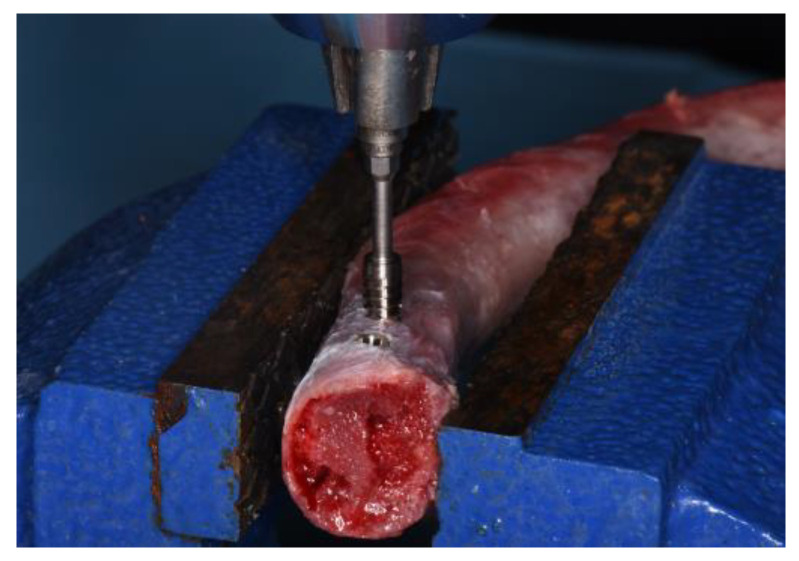
Dental implant placement and peak insertion torque recording.

Implants were manually inserted 1 mm under the level of cortical bone using the MGT-12 digital torque gauge device (Mark-10 Corp, New York, NY, USA). The increase in insertion force was 0.5 mm min^−1^, recording the peak insertion torque (PIT) in Ncm (Figure 3).

- Implant stability quotient (ISQ)

**Figure 4:**
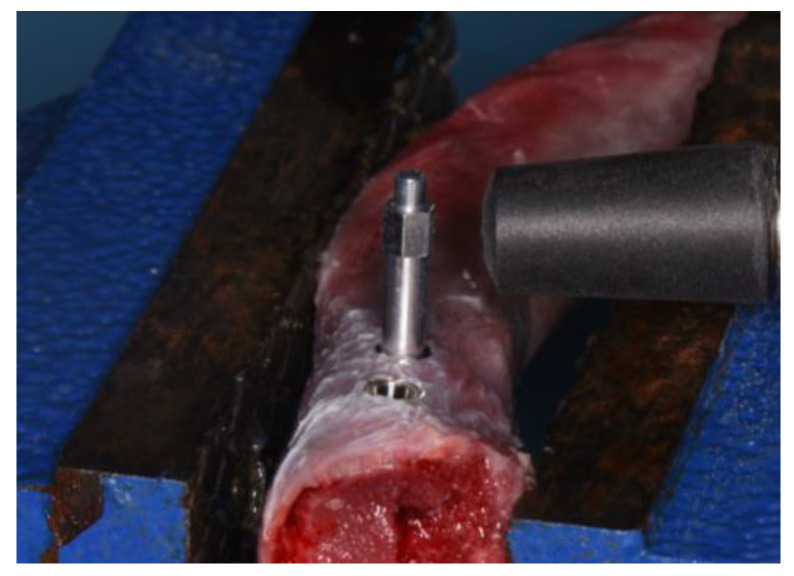
Registration of the implant stability quotient.

ISQ was recorded by performing the resonance frequency analysis (RFA) with the Osstell^®^ IDX device (W&H, Göteborg, Sweden) and by screwing in a customized SmartPeg. For each implant, RFA was performed using a no-contact technique, and two measurements of ISQ (BL and MD) were performed, directing the probe laterally in relation to the transducer (Figure 4). An independent operator repeated each measurement at least three times to validate the ISQ values, and the highest value was taken as a reference for the statistical analysis.

- Peak removal torque (PRT)/Ncm

After completing the measurements, each implant was unscrewed by the same MGT-12 digital torque device, and the peak removal torque (PRT) was recorded in Ncm.

### 2.6. Statistical Analysis

The minimum sample size was calculated for a two-tailed t-test using the software R (R version 4.3.1). The values reported in a previous study for the variable ISQ were considered for the sample size calculation [6]. It took into account the following data: mean difference = 3.65; SD = 4.085; α = 0.05; and β = 0.9. A total of 27 implants were necessary for each group. Descriptive statistics reported mean, median, and standard deviation for quantitative variables. Data analysis was conducted using the software R (R version 4.3.1). A level of *p* < 0.05 was used to indicate statistical significance.

## 3. Results

One hundred and thirty-six implants were placed into fresh pig ribs. Of these, 64 fixtures were placed using the traditional osteotomy method, following two different implant geometries, while the remaining 64 fixtures were placed using the magnetodynamic osteotomy method, also following two different implant geometries.

Results by groups are summarized in Table 1 and Table 2 in terms of mean and standard deviation.

**Figure 5:**
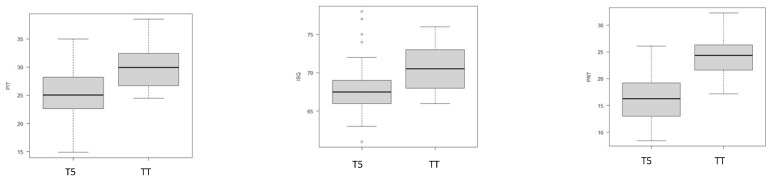
The box plots display the differences in terms of primary stability (PS) between the T5 and TT groups.

The study of the primary predictor variable showed significant differences in terms of PIT groups (*p* < 0.001), ISQ (*p* = 0.001), and PRT (*p* < 0.001) between TT and T5 groups (Figure 5).

No significant differences in primary stability parameters emerged when comparing the M5 and MT groups.

### 3.1. Primary Stability of ‘Five’ Implants Based on Surgical Procedure

The analysis of ‘Five’ macro-geometry showed how the surgical procedure can influence the results obtained for primary stability parameters.

**Figure 6:**
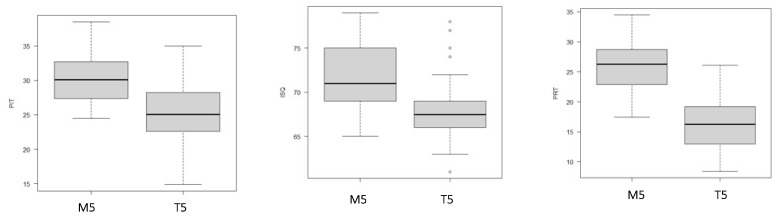
The box plots illustrate the differences in terms of primary stability (PS) between the M5 and T5 groups.

In particular, the comparison between the T5 and M5 groups revealed significant differences in PIT (*p* < 0.001), ISQ (*p* < 0.001), and PRT (*p* < 0.001) (Figure 6).

### 3.2. Primary Stability of ‘TiSmart’ Implants Based on Surgical Procedure

**Figure 7:**
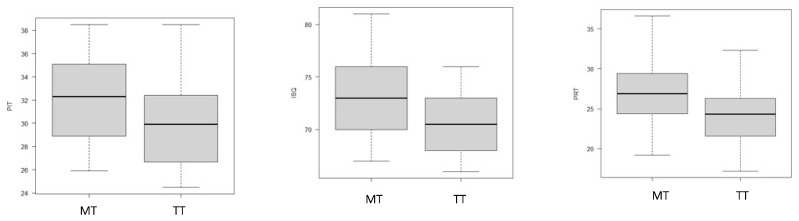
The box plots highlight the differences in primary stability parameters between the MT and TT groups.

Analyzing the macro-geometry of ‘TiSmart’ emphasized the influence of the surgical procedure on primary stability parameter outcomes. In particular, the comparison between the TT and MT groups evidenced a statistical difference in PIT (*p* = 0.02), ISQ (*p* = 0.01), and PRT (*p* < 0.001) (as shown in Figure 7).

## 4. Discussion

This in vitro study aimed to evaluate the impact of two distinct implant designs on primary implant stability in low-density bone.

In addition, the possible differences between the two implant site preparation procedures in influencing the PS values for each implant macro-design were evaluated.

Achieving good primary stability values at the time of implant placement is crucial as it helps to minimize micromovements and facilitate a strong bone-implant interface. These factors are critical in determining the appropriate loading protocols [18]. Researchers have long identified several factors that directly influence PS, such as the fixture design, the technique for the implant site osteotomy, and the quality and quantity of the bone [6,7,8,9,10]. In particular, it should be emphasized that these aspects assume an even greater significance in bone characterized by medullary tissue.

Many authors have pointed out that there is a direct correlation between bone quality and PS parameters [19,20]. The lack of primary implant stability may lead to a lower rate of osseointegration and faster peri-implant bone resorption [3].

Holahanan et al. demonstrated considerably lower survival rates for implants placed in bone of moderate/poor quality compared to those placed in predominantly dense bone [21]. Moreover, some researchers have indicated that the majority of implant failures were observed in bones of inferior quality or in upper jaws where bone resorption had resulted in a softer bone composition [22,23].

To enhance PS, various implant macro-geometries and surgical techniques were developed to improve the bone quality along the osteotomy walls [24].

Our study involved the analysis of two implants characterized by a cylindrical shape in the coronal portion and a conical shape in the central and apical parts, along with different thread profiles.

As discussed by McCullough and Klokkevold, the macro-geometry of the implant plays a key role in achieving clinical success in dental implantology. Variations in implant length, diameter, number of threads, thread depth, pitch, and helix angle can strongly influence primary stability [9].

Based on our findings, when utilizing the conventional surgical technique, our data demonstrated that the ‘TiSmart’ implant exhibited significantly greater primary stability parameter values in soft bone compared to the ‘Five’ implant. Specifically, the higher insertion torque values associated with the ‘TiSmart’ implant suggest that its variable thread design, ranging from triangular to trapezoidal, enables superior implant stability in bone with a higher proportion of marrow tissue.

These results are in agreement with the study by Menini et al. in which, for the same type of surgical preparation, implants with a triangular apical and coronal trapezoidal thread shape perform better in low-density bone, achieving better insertion torque values [25]. Many studies have analyzed how the shape of the threads can influence the primary stability of the implant; a V-shape of the threads in the apical portion of the fixture results in a greater aggressiveness of the implant, especially in low-density areas, achieving greater mechanical stability values [26,27,28]. Moreover, a trapezoidal thread design in the most coronal part of the implant also seems to induce an increased condensation of peri-implant bone during implant insertion. In low-density bones, this results in a higher impingement of the implant flange on the crestal bone [29]. The resistance of the tissue to this compression generates a rapid increase in insertion torque, resulting in better primary implant stability and, as shown in our results, higher ISQ values [30,31]. These assessments are in line with the data highlighted by Romanos et al., where through histomorphometric analysis and microradiography on animal samples, it emerged that compared to other thread geometries, progressive threads seem to provide a bone condensation effect on the surrounding bone tissue during insertion and can significantly contribute to increasing primary stability by maximizing bone-implant contacts [8].

Furthermore, the higher removal torque values reported for the ‘TiSmart’ implant seem to suggest better compaction of the coronal bone portion. Although removal torque is a parameter with poor clinical relevance, it is useful to suggest both the degree of osseointegration in ex vivo studies and to provide data on mechanical strength in in vitro studies [32].

The impact of the implant macro-geometry is further emphasized by the relevance of the fixture design in the region that comes into contact with the cortical bone. The ideal implant profile should provide a balance between compressive and tensile forces while minimizing shear force generation [33]. In fact, many studies have shown that immediately after implant placement, occlusal loads are mainly concentrated on the crestal bone-implant portion, suggesting that microthreads of the crestal portion of an implant can reduce occlusal load and contribute positively to BIC and marginal bone preservation. [26,34]. However, the detailed interplay between biological and biomechanical factors at the implant interface is not fully understood.

In our study, we observed that microthreading in the crestal portion of both implants allowed us to achieve equally favorable rates of implant stability. This was accomplished by minimizing the compressive forces on the crestal area, which are known to contribute to early loss of marginal bone and potential failure of the implant [35,36].

With the development of new technologies in the field of dentistry, researchers have come up with new choices to achieve better clinical outcomes, reduce surgical times, improve biological response, and, by considering the psychological aspect of the patient, enhance comfort and quality of life [37,38].

As previously highlighted, surgical procedures can influence PS, and with the development of new devices, many surgical techniques have been proposed to improve the quality of bone surrounding the implant site [13,24,32].

In our study, apart from examining how the two different macro implant designs affect PS, we also aimed to investigate how two distinct implant site preparation methods might influence PS values for each implant design.

In particular, we examined the method of implant site preparation using the traditional approach and the magnetodynamic approach. The statistical analysis suggested that the use of the MM for implant site preparation in low-density bone achieves significantly higher PIT, ISQ, and PRT values than traditional preparation; these in vitro data were confirmed for both macro-design ‘Five’ and ‘TiSmart’. However, there was no difference in the assessment of primary stability between the two implant shapes when the osteotomy was performed with the same magnetodynamic technique.

The Magnetic Mallet is a device that uses electromagnetic impact to produce a transient, high-intensity impact force that causes plastic deformation of bone. Compared to the traditional chisel and mallet, this device has several advantages, as it prevents the applied forces from dissipating over the entire craniofacial region [39]. The controlled fracture and displacement of bone chips and the increase in bone tissue density along the walls are the results of the longitudinal movement that the device imparts along the osteotome axis. This thrust forces the inner wall of the hole outwards from the osteotomy in a radial direction. Although several authors have exploited the magnetodynamic effect for dental extractions, currently, most studies have focused on the use of MM in implantology [40,41].

In particular, Crespi et al. conducted four clinical studies from 2012 to 2016, analyzing 218 implant site preparations using the magnetodynamic technique and reporting a survival rate of not less than 96% after 24–36 months of follow-up [41,42,43,44]. Additionally, according to histological investigations carried out by Schierano et al., it was evidenced that the use of magnetodynamic technology during implant site preparation can lead to a notable augmentation in both the quantity of newly formed bone tissue and the presence of osteoblasts, compared to the traditional drills [45]. The characteristics of this surgical approach to osteocondensate bone tissue can positively influence bone healing and primary implant stability. This statement, in agreement with our in vitro data, was confirmed by Feher et al., who evaluated the ISQ of implants placed in the condensed bone site using MM [46]. Although no significant correlations were found with insertion torque, the authors pointed out that the magnetodynamic technique resulted in higher ISQ values [46]. These findings are partially in agreement with our results, where implant site preparation performed with MM showed significantly higher PIT, ISQ, and PRT values than traditional osteotomy. However, the absence of significant differences in the PS between the two implants placed using the magnetodynamic approach suggests that this method effectively promotes bone condensation.

Moreover, in a clinical scenario, the magnetodynamic surgical procedure could be more effective in avoiding overheating of the implant site and reducing aerosol production than the traditional method, avoiding the potential spread of microorganisms into the surrounding area [39,47,48]. However, these considerations must be supported by further clinical research.

This study has all the limitations of an in vitro study, including the lack of implant osseointegration and assessment of bone-to-implant contact (BIC) at the end of the healing period. An additional limitation of the study is the lack of biological interactions between bone tissue and implants, which could highlight differences in implant site management during both early healing and prosthetic loading. Moreover, it is important to note that the absence of dedicated drills for the two distinct macro-geometries of implants could potentially affect measurements of primary implant stability. Although the study analyzed the behavior of these implant shapes and surgical techniques, an analysis of the data according to the different subtypes of low-density bone (D3-D4-D5) was not provided.

## 5. Conclusions

Our in vitro findings evidenced that both implant macro-geometries exhibit good primary stability; however, the ‘TiSmart’ shape showed better performance in low-density bone. Bone density and the relationship between the bone walls and the implant surface appear to be influenced by the implant site’s surgical preparation; specifically, the magnetodynamic procedure seems to reach higher PIT, ISQ, and PRT values than the traditional technique, regardless of the fixture geometry.

Further histological analyses conducted ex vivo could provide insights into the bone condensation potential of the magnetodynamic technique. Additionally, monitoring implant stability throughout the osseointegration period would enable a more comprehensive analysis of the two surgical techniques. The results shown by this in vitro analysis could be confirmed by further clinical studies that could highlight any advantages in the clinical performance of the two implant geometries and the two surgical approaches.

## Figures and Tables

**Table 1:** Means and SD of PIT, ISQ, and PRT of T5 group (traditional technique and ‘Five’ implant) and TT group (traditional technique and ‘TiSmart’ implant).

	T5 Group	TT Group	*p*-Value
*Peak insertion torque PIT (Ncm ± SD)*	25.04 ± 4.41	30 ± 3.74	*p* < 0.001 *
*Implant stability quotient ISQ*	68.11 ± 3.86	70.88 ± 3.08	*p* = 0.001 *
*Peak removal torque PRT (Ncm ± SD)*	16.47 ± 4.56	23.91 ± 3.28	*p* < 0.001 *

* Statistical significance (*p* < 0.05).

**Table 2:** Means and SD of PIT, ISQ, and PRT of M5 group (magnetodynamic technique and ‘Five’ implant) and MT group (magnetodynamic technique and ‘TiSmart’ implant). Ns = not significant.

	M5 Group	MT Group	*p*-Value
*Peak insertion torque PIT (Ncm ± SD)*	30.62 ± 3.81	32.05 ± 3.55	*p* = ns
*Implant stability quotient ISQ*	71.41 ± 3.69	73 ± 3.51	*p* = ns
*Peak removal torque PRT (Ncm ± SD)*	26.02 ± 4.03	26.93 ± 3.96	*p* = ns

## Data Availability

The data presented in this study are available on request from the corresponding author.
